# Sex-Based Differences in the Vitamin D Metabolite Ratio and Its Associated Factors Among Healthy Japanese Adults: A Cross-Sectional Study

**DOI:** 10.3390/biomedicines14091993

**Published:** 2026-09-04

**Authors:** Hiroyasu Miyamoto, Reiko Kisugi, Ayasa Tajima, Masaki Miyasaka, Tomokazu Matsuura, Tamayo Sano, Akihiro Kunisawa, Daisuke Kawakami, Tsuyoshi Nakanishi, Nobuhiro Hanafusa, Yutaka Furutani, Sae Ochi

**Affiliations:** 1 Department of Central Laboratory, The Jikei University Western Medical Center, Tokyo 2018601, Japan; miyamoto_h@jikei.ac.jp; 2 Department of Laboratory Medicine, The Jikei University School of Medicine, Tokyo 1050003, Japan; rkisugi@jikei.ac.jp (R.K.); a.tajima@jikei.ac.jp (A.T.); masaki108@gmail.com (M.M.); yfurutani@jikei.ac.jp (Y.F.); 3 Shonan Health Checkup Centre, Kanagawa 2540034, Japan; matsuuratomo@gmail.com; 4 Analytical and Measuring Instruments Division, Shimadzu Corporation, Kyoto 604-8511, Japan; sano.tamayo.z4w@shimadzu.co.jp (T.S.); kunisawa@shimadzu.eu (A.K.); kawakami@shimadzu.eu (D.K.); naka@shimadzu.co.jp (T.N.); hanafusa@shimadzu.co.jp (N.H.); 5 Clinical MS Solution Center, Shimadzu Europa GmbH, 47269 Duisburg, Germany

**Keywords:** 25-hydroxyvitamin D, 24,25-dihydroxyvitamin D, vitamin D metabolite ratio, sex-based difference, liquid chromatography–tandem mass spectrometry (LC-MS/MS), precision nutrition

## Abstract

**Background/Objectives**: Vitamin D is a pleiotropic substance that plays a pivotal role in the human body. Several studies have suggested sex-based differences in vitamin D metabolism. Recently, the vitamin D metabolite ratio (VMR), defined as the ratio of serum 24,25-dihydroxyvitamin D [24,25(OH)_2_D] to 25-hydroxyvitamin D [25(OH)D], has been identified as a novel marker that may more accurately reflect vitamin D status than serum 25(OH)D alone. This study aimed to assess whether significant variability exists in the VMR across different demographic groups. **Methods**: Residual serum samples and laboratory data were collected from healthy adults who underwent health checkups at a health center in Japan. Serum concentrations of 25(OH)D_3_ and 24,25(OH)_2_D_3_ were simultaneously measured using a fully automated liquid chromatography–tandem mass spectrometry (LC-MS/MS) system. Factors affecting the levels of 25(OH)D_3_, 24,25(OH)_2_D_3_, and VMR were analyzed using multiple regression analysis and the stabilized inverse probability weighting method. **Results**: Tentative reference intervals for 24,25(OH)_2_D_3_ were found to be 0.3–3.0 ng/mL for males and 0.2–2.3 ng/mL for females. Those for VMR were found to be 3.0–11.8% for males and 2.5–10.4% for females, demonstrating statistically significant but moderate differences between the sexes. These differences remained significant even after adjusting for baseline covariates including 25(OH)D_3_. However, the standard deviation ratios were less than 0.5. An estimated glomerular filtration rate and an age of ≥50 years were positively associated with 24,25(OH)_2_D_3_ and VMR, whereas diabetic status was negatively associated with these markers. **Conclusions**: This study revealed moderate sex-based differences in markers of vitamin D metabolism. Although further research is needed to determine whether these differences are consistent across other populations, future epidemiology and clinical interventions should take into consideration of the sex-based differences in vitamin D metabolism.

## 1. Introduction

Vitamin D is an essential nutrient originally identified as a nutrient that prevents rickets in children and osteomalacia in adults. Subsequent studies have demonstrated that vitamin D insufficiency is associated not only with musculoskeletal disorders but also with a wide range of conditions, including cardiovascular disease [1], infections, autoimmune diseases, depression, and infertility [2]. Vitamin D receptors (VDRs) are present in many organs throughout the body, suggesting that vitamin D insufficiency may be linked to various pathological conditions that have yet to be fully elucidated. Given its multifunctionality, accurately assessing vitamin D status has become increasingly important in clinical practice.

Vitamin D is metabolized in the liver to 25-hydroxyvitamin D [25(OH)D] and subsequently activated in the kidneys to 1,25-dihydroxyvitamin D [1,25(OH)_2_D]. Excess vitamin D is catabolized to 24,25-dihydroxyvitamin D [24,25(OH)_2_D] and excreted via the bile ducts and, to a lesser extent, the urine. Traditionally, the serum concentration of 25(OH)D, which is abundant and stable in the blood, has been used to assess vitamin D status. However, serum 25(OH)D levels are known to vary according to genetic variants [3], prompting the search for other biomarkers that enable a more precise assessment of vitamin D sufficiency/deficiency. In recent years, the development of liquid chromatography–tandem mass spectrometry (LC-MS/MS) technology has enabled us to measure the vitamin D metabolite ratio (VMR)—the ratio of serum 24,25(OH)_2_D to 25(OH)D. This ratio has been shown to be less influenced by racial differences than 25(OH)D alone [4] and to correlate more strongly with clinical outcomes, such as fracture risk [5]. Nevertheless, due to a lack of epidemiological data in healthy populations, definitive reference values for the VMR have not yet been established. It also remains unclear whether these reference values should be uniform across all ages and sexes.

Recent studies have reported sex differences in vitamin D absorption, synthesis, storage, and activation at the molecular [6] and genomic [7] levels. Additionally, sex-specific differences in the impact of low 25(OH)D levels on prediabetes [8], diabetic nephropathy and neuropathy [9], and cardiovascular disease [1] have been demonstrated. Another study revealed a sex-based difference in the threshold of serum 25(OH)D for physical function in older adults [10]. However, most of these studies used serum 25(OH)D as a marker of vitamin D sufficiency. As circulating levels of 25(OH)D are influenced by genetic variants, it is unclear whether the sex-specific differences are due to the conversion of 25(OH)D to serum, or to differences in the metabolic rate of 25(OH)D. To elucidate this mechanism, an epidemiological study examining other vitamin D metabolites is required.

Recently, epidemiological studies using VMR have been reported, revealing an association between VMR and bone mineral density [5,11], depression [12], and birth outcomes [13]. However, as many of these studies focused on patients with comorbidities such as advanced age or specific diseases, it is unclear whether sex-based differences in vitamin D metabolism exist within the cohort. Furthermore, it is also unclear whether the size of the differences in the tentative reference interval concentrations is clinically significant. Therefore, this study involved mass screening for VMR in relatively healthy Japanese adults, and the tentative sex-stratified distribution of reference intervals for metabolites was calculated to analyze whether the tentative reference intervals should be stratified by sex. Factors associated with VMR among different demographic groups were also analyzed. These findings provide novel insights into variations in vitamin D metabolism and highlight the importance of establishing precise reference intervals for vitamin D sufficiency that are tailored to patients’ background characteristics.

## 2. Materials and Methods

### 2.1. Sample Selection

Residual serum samples and health examination data were obtained from individuals undergoing health checkups at the Shimbashi Health Screening Center between April 2019 and March 2020. Pregnant or lactating women were excluded. Sixty samples were collected at random each month, except in December and March. Due to the limited number of samples available, none were obtained in December, and only fifty were collected in February. A total of 650 samples were included in the study.

### 2.2. Data Collection and Calculation

Health examination data collected included age, sex, height, weight, body mass index (BMI), fat mass, and abdominal circumference. Laboratory data included a complete blood count, albumin, aspartate aminotransferase (AST), alanine aminotransferase (ALT), gamma-glutamyl transpeptidase (γGT), pancreatic amylase (pAmy), low-density lipoprotein cholesterol (LDL-C), high-density lipoprotein cholesterol (HDL-C), triglycerides (TG), urea nitrogen (UN), creatinine (Cre), uric acid (UA), glucose, hemoglobin A1c (HbA1c). Data on serum calcium and phosphate were also collected when available (*N* = 457). The estimated glomerular filtration rate (eGFR, mL/min/1.73 m^2^) was calculated using the CKD-EPI 2021 refit equations without the race variable [14]. As these equations do not include racial adjustments, they enable a fairer international assessment of renal function. The formula is as follows:

eGFR = 142 × (Cre/A) B × 0.9938 age [×1.012 (if female)]

For males with Cre ≤ 0.9 mg/dL: A = 0.9, B= −0.302; for males with Cre > 0.9 mg/dL: A = 0.9, B = −1.2; for females with Cre ≤ 0.7 mg/dL: A = 0.7 and B = −0.241; and for females with Cre > 0.7 mg/dL: A = 0.7 and B = −1.2.

Total cholesterol (TC, mg/dL) was calculated using the Friedwald formula:

TC = LDL-C + HDL-C + TG/5.

### 2.3. Measurement of VMR

As serum 25(OH)D_2_ levels were below the detection limit in almost all samples in a previous study [15] only serum 25(OH)D_3_ and 24,25(OH)_2_D_3_ were measured using LC-MS/MS. An automated analytical system was developed, combining a fully automated preparation system (CLAM-2030; Shimadzu Corp., Kyoto, Japan), a Nexera ultra-high-performance LC system, and an ultrafast triple quadrupole mass spectrometer (LCMS-8060NX; Shimadzu Corp., Kyoto, Japan) was developed. This method was a modified version of one previously described [15].

Briefly, 90 μL of acetonitrile containing internal standards [10 ppb for 25(OH)D_3_ and 2.5 ppb for 24,25(OH)_2_D_3_] was added to 30 μL of human serum. After centrifugation at 2200 rpm for 60 s, the supernatant was filtered through a 0.45 μm polytetrafluoroethylene filter and treated with 20 μL of 75% isopropanol at −55 to −60 kPa for 60 s. A 10 μL sample was then injected into an LC-MS/MS system using electrospray ionization (ESI). To enhance sensitivity and specificity, a micellar adsorption and yttrium immobilization-octadecylsilane (MAYI 2 C18) trap column^®^ and a Shim-pack Velox PFPP analytical column^®^ (Shimadzu Corp.) were used. Analytes were identified using multiple reaction monitoring. The precursor-to-product ion transitions were *m*/*z* 383.3 → 365.3 for 25(OH)D_3_ and *m*/*z* 417.3 → 381.3 for 24,25(OH)_2_D_3_. VMR was calculated as follows:

VMR (%) = 24,25(OH)_2_D_3_/25(OH)D_3_ × 100

### 2.4. Categorization of the Variables

As most variables were not normally distributed, the following variables were categorized accordingly. Age (years) was classified as <50 and ≥50. BMI (kg/m^2^) was categorized as lean (BMI < 18.5), normal (18.5 ≤ BMI < 25), and overweight (BMI ≥ 25). HbA1c (%) was categorized as normal (<6.0), borderline (6.0 ≤ HbA1c < 6.5), and diabetes (≥6.5). Serum 25(OH)D_3_ (ng/mL) was defined as normal (≥30), insufficiency [20 ≤ 25(OH)D_3_ < 30], and deficiency (< 20 ng/mL), according to the definitions of the Japanese Society for Bone and Mineral Research and the Japan Endocrine Society [16]. A previous study showed an increased risk of femoral neck fractures at VMR values below 7% [5]; therefore, VMR < 7% was defined as low VMR.

### 2.5. Calculation of Tentative Reference Intervals and Standard Deviation Ratio

The Kolmogorov–Smirnov test was then used to assess the normality of the distribution. The *D* statistic represents the maximum vertical deviation between the empirical cumulative distribution function of the sample and the theoretical normal distribution function; non-normality was defined as *p* < 0.05. Skewed distributions were normalized using a log transformation with an optimized shift parameter. Tentative 95% reference intervals for 25(OH)D_3_, 24,25(OH)_2_D_3_, and the VMR were calculated on the transformed scale and subsequently back-transformed to the original scale. To compare the tentative reference intervals between males and females, the deviation ratio (SDR) was calculated using a formula defined by the Japanese Committee for Clinical Laboratory Standards (JCCLS) [17].

The formula is as follows:

SDR = [standard deviation (SD) between groups]/[SD between individuals (residual SD)]

Following the JCCLS guideline, SDR were interpreted as follows:

SDR < 0.3: there are small differences in the reference intervals between groups;

0.3 ≤ SDR < 0.5: there might be a difference in the reference intervals between groups; 0.5 ≤ SDR: there is a significant difference in the reference intervals between groups.

A reference interval is deemed normal for a physiological measurement in healthy individuals. However, it is difficult to find individuals who are perfectly healthy. Therefore, the tentative reference interval was calculated using data from individuals for whom all of the following were within the common reference ranges, as defined by the Japanese Committee for Clinical Laboratory Standards (JCCLS) [17]: eGFR, AST, ALT, γGT, Alb, UN, UA, LDL-C, TG, pAmy, glucose, HbA1c, white blood cell count, hemoglobin, platelet count, blood pressure, BMI, fat mass, and abdominal circumference.

### 2.6. Statistical Analyses

Sex-based differences in baseline characteristics were evaluated using a Student’s *t*-test with unequal variances. Differences between categorical variables were calculated using a chi-squared test. Multiple linear regression with robust standard errors with serum 25(OH)D_3_, 24,25(OH)_2_D_3_, and VMR as the outcomes were conducted using forced-entry models. Multicollinearity was assessed using the variance inflation factor (VIF), with VIF values greater than 5 indicating multicollinearity. Missing values were handled using multiple imputations by chained equations (MICE) under the missing at random (MAR) assumption. Twenty imputed datasets were generated using linear regression models that incorporated the dependent variable and all primary and auxiliary covariates (sex, age, eGFR, ALT, γGT, TG). The resulting estimates were then pooled using Rubin’s rules. To examine sex-specific factors associated with the vitamin D metabolites, multivariate analyses stratified by sex were also performed. Since laboratory values may vary substantially according to participant background, inverse probability weighting (IPW) based on propensity scores was applied to evaluate differences between sexes in 25(OH)D_3_, 24,25(OH)_2_D_3_ and VMR. Propensity scores were estimated using a multivariable logistic regression model. To reduce the variability of the weights and mitigate the influence of extreme propensity scores, we calculated stabilized IPW (sIPW) and fitted weighted regression models with robust standard errors to estimate the average treatment effect (ATE). In addition, to ensure robustness against potential violations of the positivity assumption, we performed a sensitivity analysis using overlap weighting. Stepwise analyses were conducted to identify the variables to be included for IPW. Significance levels of *p* > 0.2 and *p* < 0.01 were set for removal and addition to the model, respectively. Covariate balance before and after IPW was assessed using standardized mean differences (SMDs) via Stata’s tebalance command. An SMD of <0.2 was considered acceptable. Statistical significance was defined as *p* < 0.05. All analyses were performed using Stata 15.1/SE (StataCorp LLC, College Station, TX, USA).

## 3. Results

### 3.1. Validation of the LC-MS/MS Methods

The results of the validation of the accuracy of the LC-MS/MS measurements are presented in Table S1 and Figure S1. Accuracy evaluations using JEOL and NIST QC serum yielded satisfactory results, with deviations from true concentration values of less than 4% for 24,25(OH)_2_D_3_ and less than 3% for 25(OH)D_3_. The intra-day reproducibility of the NIST QC serum concentrations was 3.23% RSD for 24,25(OH)_2_D_3_ and 3.32% RSD for 25(OH)D_3_, respectively (*N* = 10). Over the course of approximately three days, more than 60 analyses were conducted, and the concentration remained stable. The standard deviation for all analyses was within 2 SD.

### 3.2. Characteristics of the Study Population

The characteristics of the study population, categorized by sex, are presented in Table 1. Serum 25(OH)D_3_, 24,25(OH)_2_D_3_, and VMR were all significantly higher in male participants than in female participants. All other variables exhibited significant differences between the groups, except for white blood cell count and total cholesterol. Overall, the prevalence of combined vitamin D insufficiency and deficiency combined was 97.4% (96.3% in males and 98.9% in females), whereas the prevalence of vitamin D deficiency alone was 72.5% (63.2% in males and 85.1% in females). The overall prevalence of a low VMR (<7%) was 65.5% (56.3% in males and 78.2% in females).

### 3.3. Tentative Reference Intervals for 24,25(OH)_2_D and VMR

**Figure 1:**
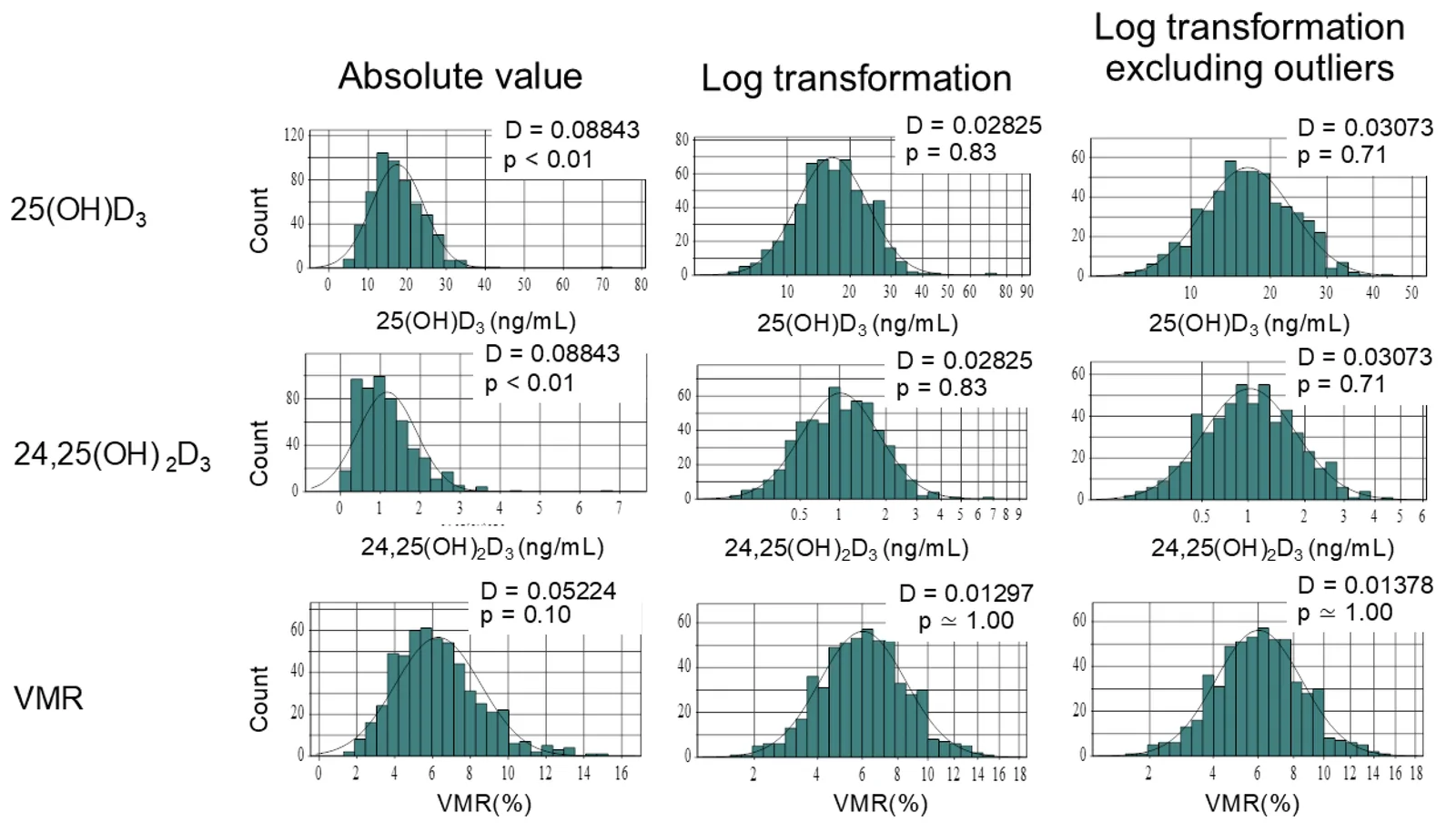
Histogram of 25(OH)D_3_, 24,25(OH)_2_D_3_, and VMR before and after transformation (*N* = 549). A Kolmogorov–Smirnov test was used to assess the skewness of the distribution.

Figure 1 shows the distribution of serum 25(OH)D_3_, 24,25(OH)_2_D_3_, and VMR. Samples for which eGFR, AST, ALT, γGT, Alb, UN, UA, LDL-C, TG, pAmy, glucose, HbA1c, white blood cell count, hemoglobin, platelet count, blood pressure, BMI, fat mass, or abdominal circumference were outside the common reference intervals were excluded. After the exclusion of these samples with extreme values, a total of 549 samples were included in the calculation.

The normality of continuous variables was assessed using the Kolmogorov–Smirnov test. Because the distributions of these variables deviated significantly from normality (all *p* < 0.05), they were log-transformed to achieve approximate normality before subsequent analyses, which was then converted back to the original scale. The median tentative reference intervals were as follows: for serum 25(OH)D_3_, 24,25(OH)_2_D_3_, and VMR were 7–32(17) ng/mL, 0.2–2.9 (1) ng/mL, and 2.7–11.4 (6.0) % in total, 9–33 (19) ng/mL, 0.3–3.0 (1.3) ng/mL, and 3.0–11.8% (6.6%) for males, and 7–29 (14) ng/mL, 0.2–2.3 (0.8) ng/mL, and 2.5–10.4% (5.35%) for females, respectively. All evaluated variables demonstrated statistically significant sex-based differences (*p* < 0.01). The SDRs for 25(OH)D_3_, 24,25(OH)_2_D_3_, and VMR were 0.44, 0.45, and 0.38, respectively. However, as the SDRs for all variables remained < 0.5, it is not strictly necessary to establish separate reference intervals for each sex (Table 2).

Restricting the cases to those with 25(OH)D levels of ≥30 ng/mL yielded 95% reference intervals of 1.87–4.49 and 5.5–11.25 for 24,25(OH)_2_D and VMR, respectively. Due to the limited number of cases, sex-based differences could not be calculated.

### 3.4. Multivariate Analyses for 25(OH)D_3_, 24,25(OH)2D_3_, and VMR

**Figure 2:**
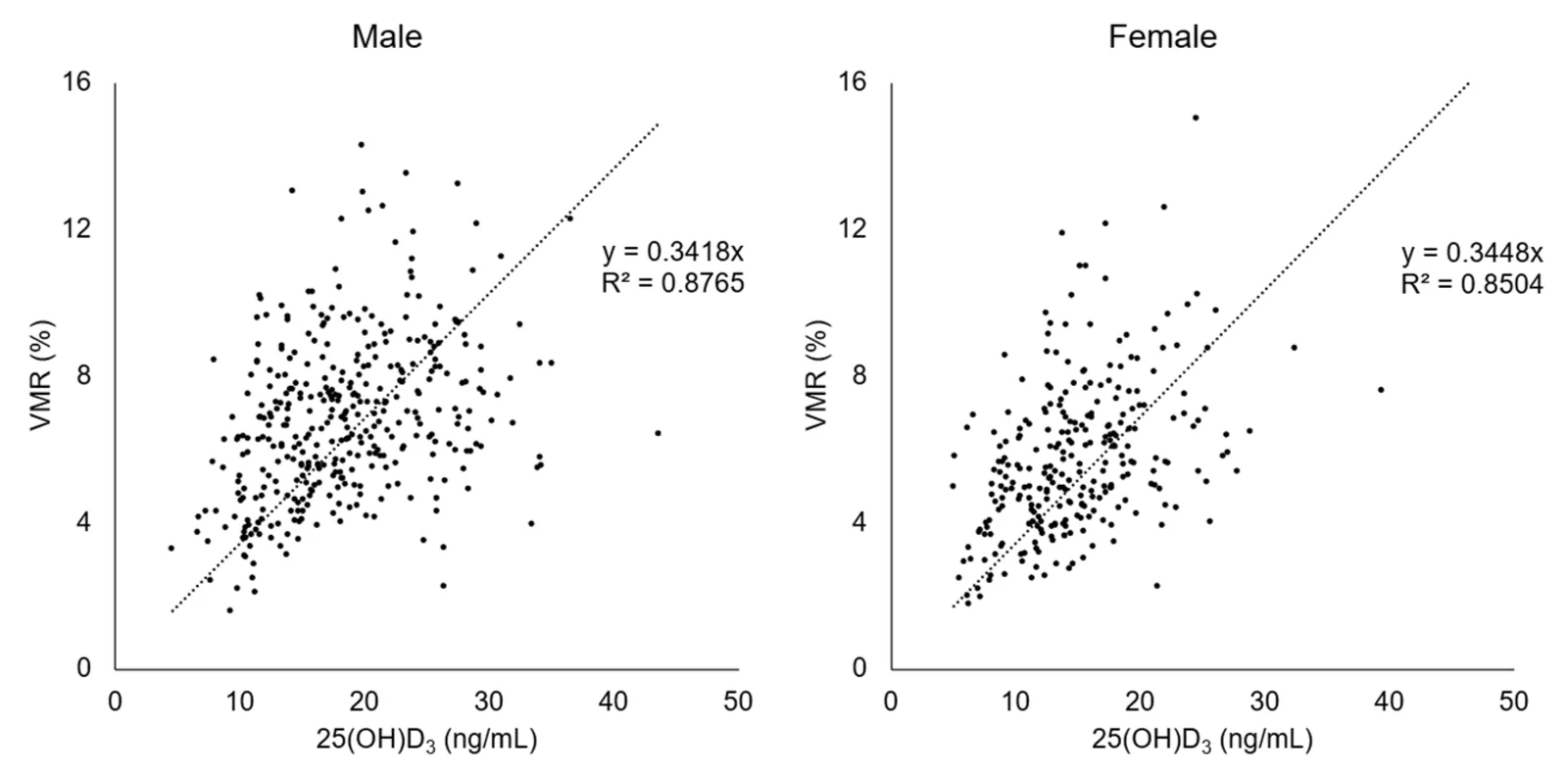
Distribution of 25(OH)D_3_ and VMR by sex for each participant.

VMR increased alongside rising serum 25(OH)D_3_ concentrations, exhibiting an approximately linear relationship (Figure 2), particularly at levels indicative of vitamin D insufficiency or deficiency. Due to the strong correlation between 25(OH)D_3_ and VMR, multivariate analyses with serum 24,25(OH)_2_D_3_ and VMR as the outcomes were conducted while controlling for serum 25(OH)D_3_ levels. Even with this adjustment, significant sex-based differences remained. Male participants exhibited higher VMR values than females. However, this difference was not observed for 24,25(OH)_2_D_3_. The coefficients of being female (95% confidence intervals [CIs]) for 25(OH)D_3_, 24,25(OH)_2_D_3_, and VMR were −3.537 (−5.572, −1.503), −0.132 (−0.250, −0.015), and −0.944 (−1.559, −0.329), respectively (Table 3). Beyond sex, an age of ≥ 50 years was positively associated with 24,25(OH)_2_D_3_ and VMR. Estimated GFR (eGFR) was positively associated with both 24,25(OH)_2_D_3_ and VMR, whereas a diabetic status (HbA1c > 6.5%) demonstrated a negative association.

Since categorizing the variables may reduce the amount of information, we also conducted the same regression analysis using continuous variables (Table S2). The results showed the same trends.

Subsequently, IPW analyses were performed while controlling for 25(OH)D_3_, age, BMI, eGFR, and HbA1c (Table 4). These controlling factors were identified by stepwise analysis for sex-based differences (Table S3). When all samples were included, the sex-based difference was statistically significant [female vs. male; average treatment effect (ATE): −0.682, 95% CI: −0.796 to −0.569 for 24,25(OH)_2_D_3_; ATE: −1.069, 95% CI: −1.616 to −0.523 for VMR]. As the SMD for 25(OH)D_3_ was greater than 0.2 (Table S3), suggesting that extreme values affected the results. Therefore, we conducted the same analysis with samples restricted to vitamin D insufficiency or deficiency. The SMDs for all variables were less than 0.2, and the difference between the sexes was significant [ATE: −0.598, 95% CI: −0.698 to −0.498 for 24,25(OH)_2_D_3_ and ATE: −1.242, 95% CI: −1.606 to −0.878 for VMR] (Table 4). The propensity score distributions of the two groups overlapped across the full range (Figure S2). The mean stabilized weight was 1.66 (standard deviation: 2.83; range: 0.45–64.78) for the total sample, and 1.47 (standard deviation: 1.07; range: 0.50–10.27) for the vitamin D insufficiency or deficiency (Table S4). Since there were potential violations of the positivity assumption, we applied the overlap weighting approach to perform a sensitivity analysis. The results confirmed the robustness of our primary findings (Table S5).

### 3.5. Differences in Associated Factors of 25(OH)D_3_, 24,25(OH)_2_D_3_ and VMR by Sex

To determine whether the factors associated with 25(OH)D_3_, 24,25(OH)_2_D_3_, and VMR differ by sex, the aforementioned multivariate analyses were stratified by sex (Table 5). Age ≥ 50 years and eGFR was positively associated with 24,25(OH)_2_D_3_ and VMR in both sexes. Diabetic status was negatively associated with 24,25(OH)_2_D_3_ and VMR in females, but not in males. Additionally, the red blood cell count was positively associated with 25(OH)D_3_ in females, while the platelet count was negatively associated with 25(OH)D_3_, 24,25(OH)_2_D_3_, and VMR in the group. When the interactions between each factor and sex were calculated, significant interactions with sex were observed in red blood cell count and diabetic status for 25(OH)D_3_, platelet count for 24,25(OH)_2_D_3_, and platelet count, total cholesterol and diabetic status for VMR.

## 4. Discussion

### 4.1. Summary of the Findings

This is the first retrospective cross-sectional study to estimate sex-specific VMR reference intervals among healthy Japanese adults. The study demonstrated statistically significant differences in markers of vitamin D metabolism, namely, serum 25(OH)D_3_, 24,25(OH)_2_D_3_, and VMR, between the sexes. Although the SDRs of the reference intervals remained <0.5, all were above 0.3, suggesting the existence of moderate sex-based differences in the intervals. Additionally, factors affecting VMR differed between the sexes, especially among those with vitamin D insufficiency/deficiency. The underlying causes of these sex-based differences may need to be elucidated to facilitate better clinical intervention.

### 4.2. Comparison with Results from Previous Studies

A previous study involving 200 healthy individuals reported similar findings: higher serum 25(OH)D and 24,25(OH)_2_D concentrations in males and non-significant trend toward a higher VMR in males [18]. Our research revealed more significant differences between the sexes, presumably due to our larger sample size. Additionally, given the strong correlation between serum 25(OH)D_3_ and 24,25(OH)_2_D_3_ concentrations, we adjusted for 25(OH)D_3_ concentration in our analyses. Another possible reason for the differences in the results is that our participants had lower baseline levels of serum 25(OH)D_3_ (17.07 ± 0.25 ng/mL) and 24,25(OH)_2_D_3_ (1.14 ± 0.03 ng/mL) than those in the previous report (24.27 ± 8.53 ng/mL and 1.93 ± 1.13 ng/mL, respectively). Since a linear correlation between 25(OH)D and VMR is primarily observed at lower 25(OH)D levels [19], the sex-based difference may be more apparent in populations with lower 25(OH)D concentrations. Indeed, the IPW analyses in our study showed that the difference was significant only under conditions of vitamin D insufficiency or deficiency (Table 4).

### 4.3. Possible Mechanisms of Sex-Based Differences

Our analyses also revealed potential sex-based differences in the factors associated with the vitamin D metabolites, such as diabetic status. This suggesting that overall vitamin D metabolism may be fundamentally influenced by sex. Vitamin D has been reported to regulate approximately 3.2 times more genes in females than in males [20], although the underlying mechanisms remain unclear. A combination of epidemiological studies and genome-wide analyses [7] may elucidate this in future.

One hypothesis is that sex hormones regulate the activity of enzymes that control vitamin D metabolism. For instance, testosterone has been shown to activate CYP27A1 and suppress CYP27B1 in vitro, which could increase VMR. Conversely estrogen may upregulate the vitamin D receptor (VDR) and enhance responsiveness to active vitamin D, thereby reducing 24,25(OH)_2_D levels and VMR [21,22]. However, these hormones may not fully explain the sex-based differences. Our study revealed that the disparity was more apparent in the older age group (≥50 years) than in the younger age group (< 50 years).

Another hypothesis is that postmenopausal status may also significantly affect vitamin D metabolism in females. For instance, bone turnover increases in postmenopausal women, resulting in increased vitamin D utilization. While our results did not demonstrate a significant impact of serum calcium and phosphate levels on the evaluated biomarkers, further analyses incorporating the measurement of parathyroid hormone (PTH) are required. Beyond calcium metabolism, postmenopausal osteoporosis is partly explained by an increase in inflammatory cytokines, such as TNF-α [23]. Our results showed a negative association between 25(OH)D_3_ levels and platelet count in females. Since platelet counts typically increase under inflammatory conditions, this result suggests a correlation between inflammation and vitamin D metabolism. However, this study did not include data on menopausal status, VDR activity, CYP enzymes, PTH, inflammatory cytokines, or bone turnover. Further research targeting patients with specific pathological conditions is necessary to elucidate the mechanisms underlying these sex-based differences in vitamin D metabolism.

### 4.4. Limitations

This research has several limitations, primarily due to its retrospective design. The most significant limitation is the lack of data on sunlight exposure, seasonal variation, and dietary/supplement intake. Seasonality data were not included in the statistical analyses, because of the limited number of samples. However, as our previous study showed, there is a difference in the serum concentration of 25(OH)D by seasons [15]. We tried to include the same number of samples for each season, but still the data lacks that in December. Second, the target population was limited to Japanese adults undergoing routine medical check-ups. Since this population excludes critically ill patients and those unable to afford medical check-ups, selection bias is unavoidable. The average height of the females in this cohort was 165.45 cm, which is taller than the national average of around 157 cm. Presumably due to this sample bias, the prevalence of vitamin D insufficiency/deficiency in our cohort is higher than that reported elsewhere [24].

Another limitation is the lack of data regarding other vitamin D- or sex-related markers, such as PTH, estrogens, and testosterone, due to constraints on the availability of residual samples. Additionally, the health checkup data did not include information on whether participants had conditions such as chronic kidney disease, diabetes, liver disease, osteoporosis, malabsorption syndrome, endocrine disease, or malignancy. We have participant-reported data on medications, but no one reported using vitamin D supplements. However, it is unclear whether participants used glucocorticoids, anticonvulsants, bisphosphonates, hormone replacement therapy, or drugs that affect CYP enzymes. Finally, although the reference intervals were calculated using data from a seemingly healthy population, these population distribution intervals differ from the thresholds for clinical outcomes and may therefore not define optimal vitamin D metabolism. Despite these limitations, the findings of this study will contribute to the field of nutrition by enhancing our understanding of sex-specific variations in the metabolism of this essential nutrient.

## 5. Conclusions

Statistically significant but moderate sex-based differences were observed in the serum concentrations of vitamin D metabolites and their associated factors in a cohort of healthy Japanese adults. These differences were more pronounced trends among older individuals and those with vitamin D insufficiency or deficiency. While these observational findings provide insight into vitamin D metabolism, prospective and interventional studies are needed to confirm whether sex-based differences affect clinical responses to vitamin D supplementation.

## Figures and Tables

**Table 1:** Background of the participants (*N* = 650). Sex-based differences were calculated using Student’s *t*-test.

Continuous Variables		Total (*N* = 650)			Male (*N* = 375)			Female (*N* = 275)			*p*
		Mean	SE	Median	Mean	SE	Median	Mean	SE	Median	
Age (year)		48.21	0.53	49	49.61	0.70	50	46.31	0.80	47	0.02
25(OH)D_3_ (ng/mL)		17.07	0.25	16.0855	18.51	0.32	17.795	15.10	0.38	14.287	<0.01
24,25(OH)_2_D_3_ (ng/mL)		1.14	0.03	0.996	1.30	0.04	1.198	0.92	0.04	0.795	<0.01
VMR (%)		6.35	0.09	6.16	6.79	0.11	6.65	5.75	0.12	5.46	<0.01
Height (cm)		166.11	0.34	166.2	166.60	0.45	167.45	165.45	0.50	165.2	0.09
Weight (kg)		63.27	0.51	61.7	64.26	0.69	63.05	61.93	0.74	60.9	0.02
BMI (kg/m^2^)		22.79	0.14	22.3	22.99	0.18	22.55	22.52	0.22	21.8	0.11
WBC (10^9^/L)		5.35	0.06	5.2	5.37	0.08	5.2	5.32	0.09	5.1	0.64
RBC (10^8^/L)		4.62	0.02	4.62	4.78	0.02	4.8	4.40	0.02	4.39	<0.01
Hb (g/dL)		14.17	0.05	14.2	14.85	0.06	14.9	13.23	0.06	13.3	<0.01
Plt (10^9^/L)		243.52	1.99	239	233.82	2.44	228	256.75	3.15	249	<0.01
AST (U/L)		23.40	0.39	21	25.61	0.57	23	20.37	0.42	19	<0.01
ALT (U/L)		22.07	0.63	17	26.54	0.92	22	15.98	0.63	13	<0.01
γGT (mg/dL)		39.12	2.16	23	52.47	3.50	32	20.92	1.09	15	<0.01
Albumin (g/dL)		4.23	0.01	4.2	4.26	0.02	4.3	4.19	0.02	4.2	<0.01
UN (mg/dL)		13.71	0.16	13	14.44	0.21	14	12.65	0.23	12	<0.01
Creatinine (mg/dL)		0.77	0.01	0.75	0.87	0.01	0.85	0.63	0.01	0.62	<0.01
UA (mg/dL)		5.44	0.05	5.4	6.11	0.06	6.2	4.51	0.06	4.5	<0.01
eGFR (ml/min/1.73 m^2)^		79.73	0.62	78.28	77.70	0.80	76.59	82.50	0.96	80.2571	<0.01
Ca (mg/dL) (*N* = 457)		9.56	0.02	9.5	9.58	0.02	9.5	9.54	0.03	9.5	0.19
iP (mg/dL) (*N* = 457)		3.56	0.02	3.6	3.45	0.03	3.4	3.72	0.03	3.7	<0.01
Total cholesterol (mg/dL)		206.03	1.37	205.4	206.36	1.83	207	205.58	2.05	202.6	0.77
LDL-Cholesterol (mg/dL)		118.88	1.21	119	121.75	1.61	122	114.95	1.81	113	<0.01
HDL-Cholesterol (mg/dL)		66.86	0.69	65.5	60.69	0.80	58	75.27	1.00	74	<0.01
Triglyceride (mg/dL)		101.49	2.78	80	119.61	4.22	95	76.79	2.48	67	<0.01
Glucose (mg/dL)		93.94	0.76	91	97.49	1.16	94	89.11	0.72	88	<0.01
HbA1c (%)		5.62	0.03	5.5	5.69	0.04	5.5	5.53	0.03	5.5	<0.01
Categorical variables		N	%		N	%		N	%		
Age category	<50 years old	347	53.4		184	49.1		163	59.3		0.01
	≥50 years old	303	46.6		191	50.9		112	40.7		
BMI category	Normal (18.5–25 kg/m^2^)	449	69.1		257	68.5		192	69.8		0.57
	Lean (<18.5 kg/m^2^)	55	8.5		29	7.7		26	9.5		
	Overweight (>25 kg/m^2^)	145	22.3		88	23.5		57	20.7		
	Missing data	1	0.2		1	0.3		0	0.0		
25(OH)D_3_ status	Normal (≥30 ng/mL)	17	2.6		14	3.7		3	1.1		<0.01
	Insufficiency (≥20, <30 ng/mL)	162	24.9		124	33.1		38	13.8		
	Deficiency (<20 ng/mL)	471	72.5		237	63.2		234	85.1		
VMR level	Normal (≥7%)	224	34.5		164	43.7		60	21.8		<0.01
	Low (<7%)	426	65.5		211	56.3		215	78.2		
HbA1c status	Normal (<6.0%)	563	86.6		309	82.4		254	92.4		<0.01
	Border (≥6.0, <6.5%)	52	8.0		39	10.4		13	4.7		
	DM (≥6.5%)	35	5.4		27	7.2		8	2.9		

SE: standard error, 25(OH)D: 25 hydroxyvitamin D, 24,25(OH)_2_D: 24,25-dihydroxyvitamin D, VMR: vitamin D metabolite ratio, BMI: body mass index, WBC: white blood cell count, RBC: red blood cell count, Hb: hemoglobin, Plt: platelet count, AST: aspartate aminotransferase, ALT: alanine aminotransferase, γGT: gamma-glutamyl transpeptidase, UN: urea nitrogen, UA: uric acid, eGFR: estimated glomerular filtration rate, Ca: calcium, iP: inorganic phosphate, LDL: low-density lipoprotein, HDL: high-density lipoprotein, DM: diabetes mellitus.

**Table 2:** Tentative reference intervals of 25(OH)D_3_, 24,25(OH)_2_D_3_, and VMR by sex. Sex-based differences were calculated using Student’s *t*-test. The calculation used data from individuals for whom the following were all within the common reference ranges as defined by the Japanese Committee for Clinical Laboratory Standards (JCCLS) [17]: eGFR, AST, ALT, γGT, Alb, UN, UA, LDL-C, TG, pAmy, glucose, HbA1c, white blood cell count, hemoglobin, platelet count, blood pressure, BMI, fat mass, and abdominal circumference.

	Total (*N* = 549)				Male (*N* = 309)				Female (*N* = 240)				p	SDR
	Tentative 95% Reference Interval			Median	Tentative 95% Reference Interval			Median	Tentative 95% Reference Interval			Median		
25(OH)D_3_ (ng/mL)	7	-	32	17	9	-	33	19	7	-	29	14	<0.01	0.44
24,25(OH)_2_D_3_ (ng/mL)	0.2	-	2.9	1	0.3	-	3.0	1.3	0.2	-	2.3	0.8	<0.01	0.45
VMR (%)	2.7	-	11.4	6.0	3.0	-	11.8	6.6	2.5	-	10.4	5.35	<0.01	0.38

25(OH)D: 25 hydroxyvitamin D, 24,25(OH)_2_D: 24,25-dihydroxyvitamin D, VMR: vitamin D metabolite ratio.

**Table 3:** Multiple regression analyses for the factors associated with 25(OH)D_3_, 24,25(OH)_2_D_3_, and VMR (*N* = 649).

		25(OH)D_3_				24,25(OH)_2_D_3_				VMR			
		Coefficient	95% CI		*p*	Coefficient	95% CI		*p*	Coefficient	95% CI		*p*
25(OH)D_3_ (ng/mL)		N.A.				0.085	0.077	0.094	<0.01	0.122	0.081	0.163	<0.01
Sex	Male	0 (Reference)				0 (Reference)				0 (Reference)			
	Female	−3.537	−5.572	−1.503	<0.01	−0.132	−0.250	−0.015	0.03	−0.944	−1.559	−0.329	<0.01
Age category	<50 years old	0 (Reference)				0 (Reference)				0 (Reference)			
	≥50 years old	−1.668	−3.304	−0.033	0.05	0.181	0.064	0.298	<0.01	1.020	0.451	1.590	<0.01
BMI category	Normal (18.5–25 kg/m^2^)	0 (Reference)				0 (Reference)				0 (Reference)			
	Lean (<18.5 kg/m^2^)	1.453	−1.233	4.139	0.29	0.077	−0.078	0.232	0.33	0.583	−0.244	1.410	0.17
	Overweight (>25 kg/m^2^)	−0.138	−1.614	1.338	0.85	−0.121	−0.235	−0.007	0.04	−0.549	−1.102	0.005	0.05
WBC (10^9^/L)		−0.458	−0.988	0.072	0.09	−0.006	−0.043	0.030	0.73	−0.054	−0.231	0.123	0.55
RBC (10^8^/L)		0.899	−1.423	3.221	0.45	0.008	−0.156	0.172	0.92	−0.016	−0.779	0.747	0.97
Plt (10^9^/L)		−0.008	−0.022	0.006	0.24	0.000	−0.001	0.000	0.30	−0.003	−0.008	0.002	0.23
Albumin (g/dL)		−1.108	−4.517	2.302	0.52	0.046	−0.168	0.260	0.67	0.398	−0.669	1.464	0.46
eGFR (ml/min/1.73 m^2^)		−0.072	−0.148	0.003	0.06	0.008	0.003	0.013	<0.01	0.035	0.012	0.057	<0.01
UA (mg/dL)		0.189	−0.481	0.859	0.58	0.014	−0.033	0.062	0.55	−0.024	−0.242	0.194	0.83
ALT (U/L)		−0.003	−0.066	0.060	0.92	−0.002	−0.005	0.002	0.40	−0.009	−0.026	0.007	0.28
γGT (mg/dL)		0.013	0.002	0.025	0.02	0.001	0.000	0.003	0.03	0.003	0.000	0.007	0.05
Total cholesterol (mg/dL)		0.024	0.002	0.045	0.03	0.000	−0.001	0.002	0.59	0.001	−0.007	0.009	0.77
Triglyceride (mg/dL)		0.001	−0.011	0.014	0.82	−0.001	−0.001	0.000	0.19	−0.002	−0.006	0.002	0.35
Ca (mg/dL)		2.754	−0.111	5.620	0.06	−0.040	−0.187	0.106	0.59	−0.261	−1.000	0.477	0.49
iP (mg/dL)		0.429	−1.181	2.039	0.60	0.061	−0.036	0.159	0.22	0.268	−0.232	0.769	0.29
HbA1c status	Normal (<6.0%)	0 (Reference)				0 (Reference)				0 (Reference)			
	Border (≥6.0%, <6.5%)	−0.261	−2.195	1.674	0.79	0.031	−0.120	0.182	0.69	0.387	−0.396	1.171	0.33
	DM (≥6.5%)	−2.664	−5.864	0.537	0.10	−0.215	−0.358	−0.073	<0.01	−1.138	−1.918	−0.358	<0.01

25(OH)D: 25 hydroxyvitamin D, 24,25(OH)_2_D: 24,25-dihydroxyvitamin D, VMR: vitamin D metabolite ratio, BMI: body mass index, WBC: white blood cell count, RBC: red blood cell count, Plt: platelet count, UA: uric acid, eGFR: estimated glomerular filtration rate, ALT: alanine aminotransferase, γGT: gamma-glutamyl transpeptidase, Ca: calcium, iP: inorganic phosphate, DM: diabetes mellitus, N.A.: not applicable.

**Table 4:** Stabilized inverse probability weighting (sIPW) analysis for sex-based difference in 24,25(OH)_2_D_3_ and VMR. The analysis was conducted on the total samples (*N* = 649) and on samples with vitamin D insufficiency or deficiency [serum 25(OH)D_3_ < 30 ng/mL] (*N* = 632), controlling for 25(OH)D_3_, age, BMI, eGFR, WBC, and HbA1c. One sample lacked the data of BMI and it was excluded from the analyses.

		ATE (Female vs. Male)	95% CI		*p*
24,25(OH)_2_D_3_	Total *(N* = 649)	−0.682	−0.796	−0.569	<0.01
	Vitamin D insufficiency/deficiency (*N* = 632)	−0.598	−0.698	−0.498	<0.01
VMR	Total (*N* = 649)	−1.069	−1.616	−0.523	<0.01
	Vitamin D insufficiency/deficiency (*N* = 632)	−1.242	−1.606	−0.878	<0.01

ATE: average treatment effect; CI: confidence interval; 24,25(OH)_2_D: 24,25-dihydroxyvitamin D; VMR: vitamin D metabolite ratio.

**Table 5:** Multiple regression analysis for the factors associated with 25(OH)D_3_, 24,25(OH)_2_D_3_, and VMR by sex.

25(OH)D_3_										
		**Male (*N* = 374)**				**Female (*N* = 275)**				***p*-Values of Interaction**
		**Coefficient**	**95%CI**		*p*	**Coefficient**	**95%CI**		*p*	
Age	<50 years	0 (Reference)				0 (Reference)				
	≥50 years	−1.239	−3.709	1.231	0.32	−1.793	−4.021	0.434	0.11	0.74
BMI category	Normal (18.5–25 kg/m^2^)	0 (Reference)				0 (Reference)				
	Lean (<18.5 kg/m^2^)	0.731	−2.851	4.313	0.69	2.787	−1.182	6.757	0.17	0.45
	Overweight (>25 kg/m^2^)	−0.199	−2.151	1.752	0.84	0.162	−2.516	2.840	0.91	0.83
WBC (10^9^/L)		−0.374	−1.155	0.407	0.35	−0.241	−0.966	0.483	0.51	0.81
RBC (10^8^/L)		−0.295	−3.379	2.789	0.85	5.305	2.532	8.078	<0.01	0.01
Plt (10^9^/L)		−0.002	−0.022	0.019	0.88	−0.023	−0.044	−0.002	0.03	0.15
Albumin (g/dL)		−1.525	−6.512	3.461	0.55	−1.026	−5.682	3.630	0.66	0.89
eGFR (ml/min/1.73 m^2)^		−0.062	−0.157	0.033	0.20	−0.090	−0.237	0.057	0.23	0.75
UA (mg/dL)		0.301	−0.528	1.130	0.47	0.002	−1.269	1.272	1.00	0.69
ALT (U/L)		0.006	−0.066	0.079	0.86	−0.032	−0.129	0.065	0.51	0.53
γGT (mg/dL)		0.012	−0.001	0.025	0.07	−0.010	−0.079	0.059	0.77	0.53
Total cholesterol (mg/dL)		0.032	0.000	0.064	0.05	0.019	−0.011	0.048	0.21	0.54
Triglyceride (mg/dL)		0.001	−0.014	0.016	0.87	−0.021	−0.050	0.008	0.16	0.18
Ca (mg/dL)		3.049	−1.010	7.108	0.14	1.533	−1.703	4.770	0.35	0.56
iP(mg/dL)		1.212	−1.141	3.564	0.31	−0.845	−3.532	1.842	0.53	0.25
HbA1c status	Normal (<6.0%)	0 (Reference)				0 (Reference)				
	Border (≥6.0, <6.5%)	−0.512	−3.132	2.108	0.70	1.402	−1.442	4.246	0.33	0.33
	DM (≥6.5%)	−3.957	−7.004	−0.910	0.01	3.588	−1.302	8.478	0.15	0.01
24,25(OH)_2_D_3_										
		Male (*N* = 374)				Female (*N* = 275)				*p*-Values of Interaction
		Coefficient	95%CI		*p*	Coefficient	95%CI		*p*	
25(OH)D_3_ (ng/mL)		0.087	0.075	0.099	<0.01	0.079	0.066	0.092	<0.01	0.38
Age	<50 years	0 (Reference)				0 (Reference)				
	≥50 years	0.180	0.004	0.357	0.05	0.161	0.016	0.306	0.03	0.87
BMI category	Normal (18.5–25 kg/m^2^)	0 (Reference)				0 (Reference)				
	Lean (<18.5 kg/m^2^)	0.103	−0.087	0.293	0.29	−0.002	−0.256	0.251	0.99	0.51
	Overweight (>25 kg/m^2^)	−0.125	−0.286	0.036	0.13	−0.164	−0.331	0.002	0.05	0.74
WBC (10^9^/L)		0.000	−0.056	0.056	0.99	0.004	−0.042	0.050	0.86	0.91
RBC (10^8^/L)		−0.004	−0.227	0.218	0.97	0.109	−0.104	0.322	0.31	0.47
Plt (10^9^/L)		0.000	−0.001	0.002	0.59	−0.002	−0.003	0.000	0.01	0.03
Albumin (g/dL)		0.006	−0.325	0.337	0.97	0.170	−0.092	0.431	0.20	0.44
eGFR (ml/min/1.73 m^2^)		0.008	0.001	0.015	0.03	0.007	0.001	0.012	0.03	0.80
UA (mg/dL)		0.028	−0.035	0.090	0.39	−0.010	−0.076	0.055	0.75	0.40
ALT (U/L)		−0.002	−0.007	0.003	0.41	−0.001	−0.005	0.004	0.78	0.70
γGT (mg/dL)		0.001	0.000	0.003	0.02	0.000	−0.003	0.003	0.99	0.35
Total cholesterol (mg/dL)		0.002	0.000	0.005	0.10	−0.001	−0.003	0.001	0.29	0.05
Triglyceride (mg/dL)		−0.001	−0.002	0.000	0.08	0.000	−0.002	0.001	0.72	0.49
Ca (mg/dL)		−0.144	−0.374	0.087	0.22	0.045	−0.124	0.213	0.60	0.19
iP(mg/dL)		0.083	−0.074	0.240	0.30	0.117	−0.013	0.248	0.08	0.74
HbA1c status	Normal (<6.0%)	0 (Reference)				0 (Reference)				
	Border (≥6.0, <6.5%)	0.058	−0.131	0.248	0.55	0.014	−0.260	0.288	0.92	0.79
	DM (≥6.5%)	−0.102	−0.278	0.073	0.25	−0.357	−0.578	−0.136	<0.01	0.07
VMR										
		Male (*N* = 374)				Female (*N* = 275)				*p*-Values of Interaction
		Coefficient	95%CI		*p*	Coefficient	95%CI		*p*	
25(OH)D_3_ (ng/mL)		0.100	0.048	0.152	<0.01	0.149	0.079	0.220	<0.01	0.26
Age	<50 years	0 (Reference)				0 (Reference)				
	≥50 years	1.085	0.276	1.894	0.01	0.943	0.123	1.763	0.03	0.81
BMI category	Normal (18.5–25 kg/m^2^)	0 (Reference)				0 (Reference)				
	Lean (<18.5 kg/m^2^)	0.539	−0.349	1.428	0.23	0.319	−1.283	1.921	0.69	0.81
	Overweight (>25 kg/m^2^)	−0.505	−1.239	0.230	0.18	−0.829	−1.759	0.100	0.08	0.59
WBC (10^9^/L)		0.020	−0.239	0.280	0.88	−0.091	−0.338	0.156	0.47	0.54
RBC (10^8^/L)		−0.112	−1.095	0.871	0.82	0.384	−0.747	1.515	0.50	0.51
Plt (10^9^/L)		0.002	−0.004	0.009	0.47	−0.008	−0.014	−0.001	0.03	0.03
Albumin (g/dL)		0.166	−1.380	1.712	0.83	0.785	−0.659	2.230	0.28	0.56
eGFR (ml/min/1.73 m^2^)		0.033	0.003	0.062	0.03	0.035	0.000	0.070	0.05	0.92
UA (mg/dL)		0.051	−0.228	0.330	0.72	−0.067	−0.435	0.300	0.72	0.61
ALT (U/L)		−0.011	−0.033	0.010	0.30	−0.001	−0.028	0.025	0.92	0.56
γGT (mg/dL)		0.004	0.000	0.008	0.07	0.007	−0.009	0.024	0.38	0.69
Total cholesterol (mg/dL)		0.010	−0.001	0.021	0.08	−0.006	−0.018	0.006	0.31	0.05
Triglyceride (mg/dL)		−0.003	−0.008	0.001	0.17	−0.004	−0.014	0.006	0.39	0.86
Ca (mg/dL)		−0.700	−1.794	0.394	0.21	0.073	−0.828	0.973	0.87	0.28
iP (mg/dL)		0.551	−0.211	1.312	0.16	0.230	−0.445	0.905	0.50	0.53
HbA1c status	Normal (<6.0%)	0 (Reference)				0 (Reference)				
	Border (≥6.0, <6.5%)	0.423	−0.472	1.318	0.35	0.381	−1.454	2.215	0.68	0.97
	DM (≥6.5%)	−0.662	−1.651	0.326	0.19	−2.238	−3.175	−1.300	<0.01	0.02

25(OH)D: 25 hydroxyvitamin D, 24,25(OH)_2_D: 24,25-dihydroxyvitamin D, VMR: vitamin D metabolite ratio, BMI: body mass index, WBC: white blood cell count, RBC: red blood cell count, Plt: platelet count, UA: uric acid, eGFR: estimated glomerular filtration rate, ALT: alanine aminotransferase, γGT: gamma-glutamyl transpeptidase, Ca: calcium, iP: inorganic phosphate, DM: diabetes mellitus.

## Data Availability

Data is available on request due to contraction issue.

## Supplementary Materials

The following supporting information can be downloaded at: https://www.mdpi.com/article/10.3390/biomedicines14091993/s1, Figure S1: Inter-day reproducibility of the LC-MS/MS system; Table S2: Multiple regression analyses using continuous variables; Table S3: Standard mean differences (SMDs) of the IPW analyses in Table 4; Figure S2: Distribution of propensity scores by sex. After the IPW analyses in Table 4. Table S1: Validation results of accuracy of the LC-MS/MS system; Table S4: Distribution of mean stabilized weight in the IPW analyses in Table 4; Table S5: IPW with an overlapping weighing approach. The confounding factors used are the same as in Table 4.

## Abbreviations

The following abbreviations are used in this manuscript:

1,25(OH)_2_D	1,25-dihydroxyvitamin D
24,25(OH)_2_D	24,25-dihydroxyvitamin D
25(OH)D	25-hydroxyvitamin D
ALT	Alanine aminotransferase
AST	Aspartate aminotransferase
ATE	Average treatment effect
BMI	Body mass index
CI	Confidence interval
Cr	Creatinine
DM	Diabetes mellitus
eGFR	Estimated glomerular filtration rate
HbA1c	Hemoglobin A1c
HDL-C	High-density lipoprotein cholesterol
IPW	Inverse probability weighting
γGT	Gamma-glutamyl transpeptidase
LDL-C	Low-density lipoprotein cholesterol
pAmy	Pancreatic amylase
Plt	Platelet
RBC	Red blood cell
SD	Standard deviation
SDR	Standard deviation ratio
SE	Standard error
SMD	Standardized mean difference
TG	Triglycerides
UN	Urea nitrogen
UA	Uric acid
VDR	Vitamin D receptor
VIF	Variance inflation factor
WBC	White blood cell
