# Supplementary material for: Sex-Based Differences in the Vitamin D Metabolite Ratio and Its Associated Factors Among Healthy Japanese Adults: A Cross-Sectional Study
Source: Biomedicines. 2026 Sep 4;14(9):1993. doi: 10.3390/biomedicines14091993 (PMC13604202; doi:10.3390/biomedicines14091993)
Supplement: Supplementary file 1 [file biomedicines-14-01993-s001.zip › biomedicines-4455255-supplementary.pdf]

Table S1. Validation results of accuracy of the LC-MS/MS system.

	$25(OH)D_3$			$24,25(OH)_2D_3$		
	Measured Concentration (ng/mL)	Nominal Concentration (ng/mL)	Accuracy (%)	Measured Concentration (ng/mL)	Nominal Concentration (ng/mL)	Accuracy (%)
JEOL Calibrator	0.88	0.88	100.1	0.094	0.094	100.3
	8.75	8.78	99.6	0.91	0.95	95.9
	41	43.9	93.4	4.74	4.75	99.7
	93.9	87.8	106.9	9.87	9.49	104.2
NIST QC	29	29.4	98.7	2.69	2.64	101.8
JEOL QC	25.1	25.7	97.8	0.55	0.54	103.2

Figure S1. Inter-day reproducibility of the LC-MS/MS system

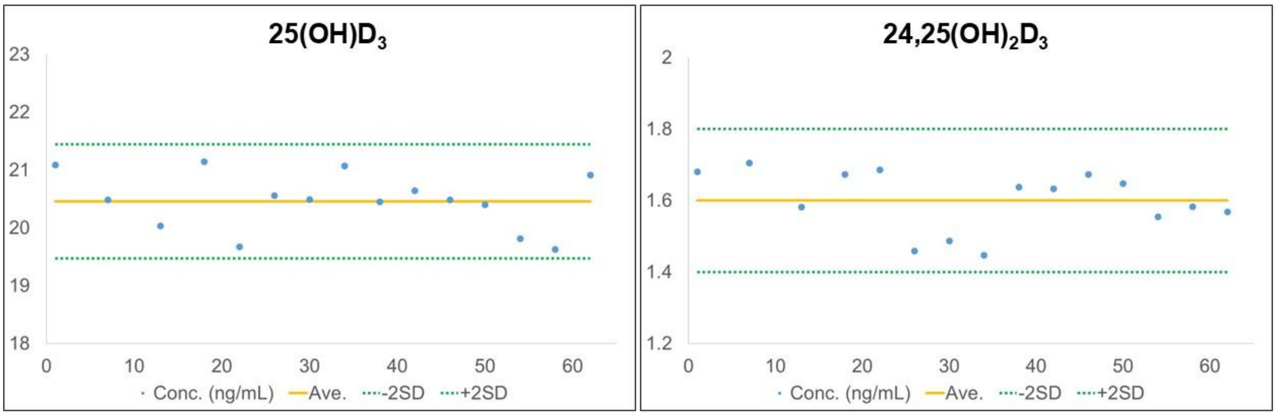

Conc: concentration, Ave: average titer, SD: standard deviation

Table S2. Multiple regression analyses using continuous variables

	$25(OH)D_3$ (ng/mL)				$24{,}25(OH)_2D_3$ (ng/mL)				VMR (%)			
	Coefficient	95%CI		p	Coefficient	95%CI		p	Coefficient	95%CI		p
$25(OH)D_3$ (ng/mL)		N.A.			0.08	0.08	0.09	< 0.01	0.12	0.07	0.16	< 0.01
Sex(female)	-3.49	-5.52	-1.47	< 0.01	-0.15	-0.26	-0.03	0.02	-1.02	-1.64	-0.40	< 0.01
Age (year)	-0.02	-0.11	0.07	0.66	0.01	0.00	0.01	0.03	0.04	0.01	0.07	0.01
BMI (kg/m^2)	-0.09	-0.27	0.08	0.30	-0.01	-0.03	0.00	0.05	-0.07	-0.14	-0.01	0.03
WBC (10^9/L)	-0.46	-0.99	0.08	0.09	-0.01	-0.05	0.03	0.56	-0.08	-0.26	0.10	0.40
RBC (10^8/L)	1.19	-1.18	3.55	0.32	0.03	-0.13	0.18	0.75	0.08	-0.65	0.81	0.83
Plt (10^9/L)	-0.01	-0.02	0.01	0.28	0.00	0.00	0.00	0.33	0.00	-0.01	0.00	0.24
Albumin (g/dL)	-0.98	-4.36	2.41	0.57	0.01	-0.22	0.23	0.95	0.14	-0.98	1.25	0.81
eGFR (ml/min/1.73m$^{2)}$	-0.06	-0.14	0.03	0.20	0.01	0.00	0.01	0.01	0.03	0.01	0.06	0.01
UA (mg/dL)	0.26	-0.43	0.94	0.46	0.01	-0.03	0.06	0.60	-0.03	-0.25	0.19	0.78
ALT (U/L)	-0.01	-0.07	0.06	0.86	0.00	0.00	0.00	0.42	-0.01	-0.02	0.01	0.22
γGT (mg/dL)	0.01	0.00	0.02	0.06	0.00	0.00	0.00	0.01	0.00	0.00	0.01	0.02
Total cholesterol (mg/dL)	0.02	0.00	0.04	0.03	0.00	0.00	0.00	0.20	0.00	0.00	0.01	0.25
Total cholesterol (mg/dL)	0.00	-0.01	0.01	0.78	0.00	0.00	0.00	0.16	0.00	-0.01	0.00	0.32
Ca (mg/dL)	-1.24	-2.82	0.34	0.12	-0.12	-0.21	-0.04	0.01	-0.56	-1.02	-0.09	0.02
iP (mg/dL)	2.62	-0.18	5.42	0.07	-0.03	-0.18	0.12	0.67	-0.21	-0.95	0.53	0.57
HbA1c (%)	0.44	-1.16	2.05	0.59	0.07	-0.03	0.17	0.15	0.33	-0.18	0.83	0.21

25(OH)D: 25 hydroxyvitamin D, $24{,}25(OH)_2D$: 24,25-dihydroxyvitamin D, VMR: vitamin D metabolite ratio, BMI: body mass index, WBC: white blood cell count, RBC: red blood cell count, Plt: platelet count, UA: uric acid, eGFR: estimated glomerular filtration rate, ALT: alanine aminotransferase, γ GT: gamma-glutamyl transpeptidase, Ca: calcium, iP: inorganic phosphate, N.A.: not applicable

Figure S2. Distribution of propensity scores by sex.

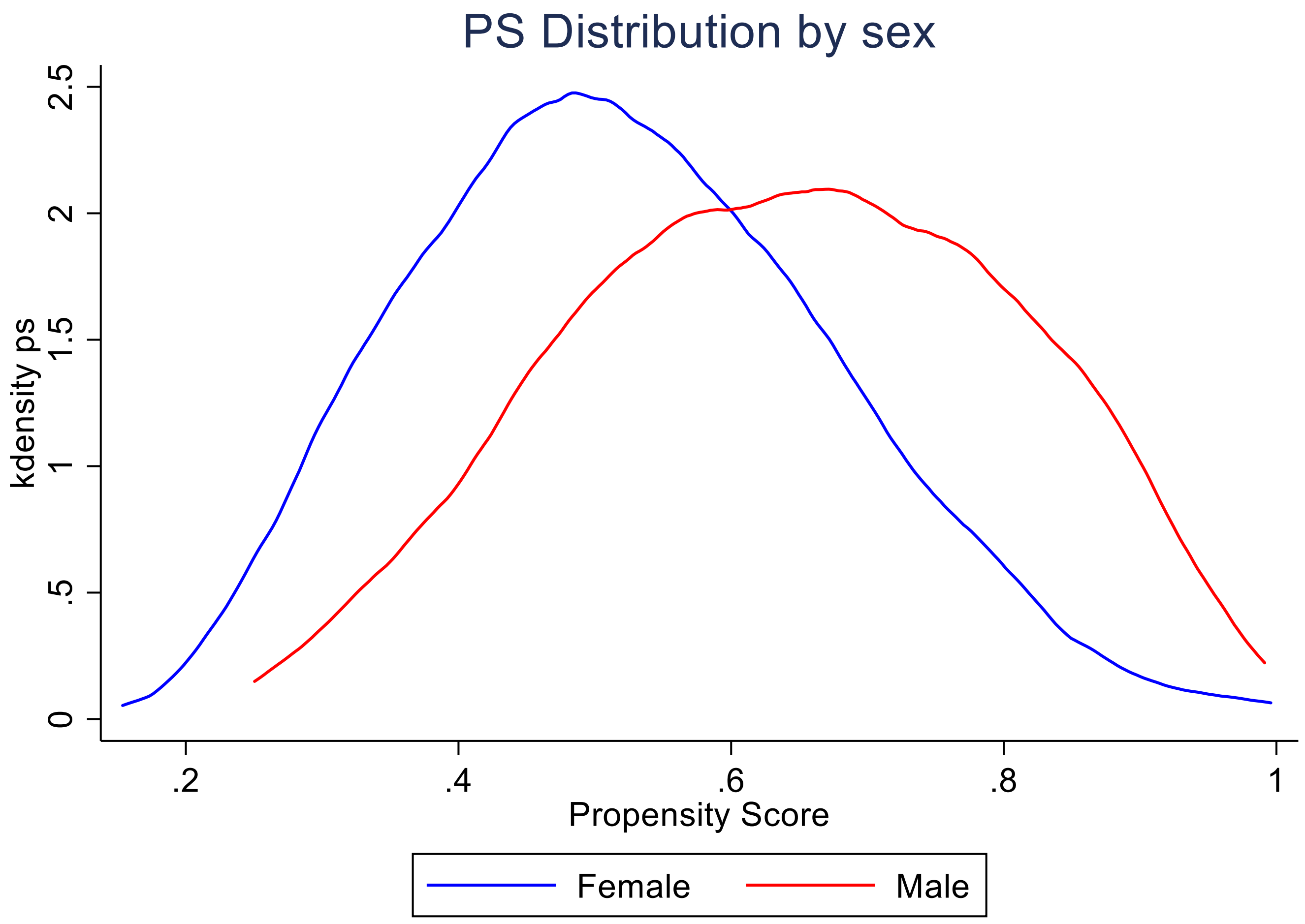

Table S3. Standard mean differences (SMDs) of the IPW analyses in Table 4.

	$24,25(OH)_2D$		VMR	
	Raw	Weighted	Raw	Weighted
Total				
25(OH)D	-0.54	0.82	-0.54	0.82
Age	-0.25	-0.21	-0.25	-0.21
BMI	-0.13	-0.02	-0.13	-0.02
eGFR	0.48	-0.14	0.48	-0.14
WBC	-0.04	0.10	-0.04	0.10
HbA1c	-0.24	-0.08	-0.24	-0.08
Vitamin D insufficiency/deficiency				
25(OH)D	-0.86	0.10	-0.86	0.10
Age	-0.08	0.05	-0.08	0.05
BMI	-0.51	0.12	-0.51	0.12
eGFR	0.24	0.00	0.24	0.00
WBC	-0.25	0.06	-0.25	0.06
HbA1c	-0.34	0.13	-0.34	0.13

25(OH)D: 25 hydroxyvitamin D, $24,25(OH)_2D$: 24,25-dihydroxyvitamin D, VMR: vitamin D metabolite ratio, BMI: body mass index, WBC: white blood cell count, eGFR: estimated glomerular filtration rate, HbA1c: hemoglobin A1c.

Table S4. Distribution of mean stabilized weight in the IPW analyses in Table 4.

	N	Mean	SD	Minimum	1 percentile	5 percentile	50 percentile	95 percentile	99 percentile	Maximum
**Total**	649	1.66	2.83	0.45	0.54	0.64	1.17	3.89	7.69	64.78
**Vitamin D insufficiency/deficiency**	632	1.48	1.08	0.50	0.55	0.65	1.17	3.39	6.37	10.27

Table S5. IPW with an overlapping weighing approach. The confounding factors used are the same as in Table 4.

		ATE (female vs male)	95% CI		p
$24,25(OH)_2D_3$	Total	-0.597	-0.692	-0.503	<0.01
	Vitamin D insufficiency/deficiency	-0.536	-0.625	-0.446	<0.01
VMR	Total	-1.302	-1.638	-0.966	<0.01
	Vitamin D insufficiency/deficiency	-1.269	-1.608	-0.930	<0.01

ATE: average treatment effect; CI: confidence interval; $24,25(OH)_2D$: 24,25-dihydroxyvitamin D; VMR: vitamin D metabolite ratio
